# Abcb4-defect cholangitis mouse model with hydrophobic bile acid composition by *in vivo* liver-specific gene deletion

**DOI:** 10.1016/j.jlr.2024.100616

**Published:** 2024-08-05

**Authors:** Kota Tsuruya, Keiko Yokoyama, Yusuke Mishima, Kinuyo Ida, Takuma Araki, Satsuki Ieda, Masato Ohtsuka, Yutaka Inagaki, Akira Honda, Tatehiro Kagawa, Akihide Kamiya

**Affiliations:** 1 Division of Gastroenterology, Department of Internal Medicine, Tokai University School of Medicine, Isehara, Kanagawa, Japan; 2 Department of Molecular Life Sciences, Tokai University School of Medicine, Isehara, Kanagawa, Japan; 3 Support Center of Medical Research and Education, Tokai University School of Medicine, Isehara, Kanagawa, Japan; 4 Center for Matrix Biology and Medicine, Tokai University School of Medicine, Isehara, Kanagawa, Japan; 5 Joint Research Center, Tokyo Medical University Ibaraki Medical Center, Ibaraki, Japan; 6 Department of Gastroenterology and Hepatology, Tokyo Medical University Ibaraki Medical Center, Ibaraki, Japan

**Keywords:** progressive familial intrahepatic cholestasis, genome editing, bile acid composition, adeno-associated virus

## Abstract

Progressive familial intrahepatic cholestasis (PFIC) is a liver disease that occurs during childhood and requires liver transplantation. ABCB4 is localized along the canalicular membranes of hepatocytes, transports phosphatidylcholine into bile, and its mutation causes PFIC3. Abcb4 gene-deficient mice established as animal models of PFIC3 exhibit cholestasis-induced liver injury. However, their phenotypes are often milder than those of human PFIC3, partly because of the existence of large amounts of less toxic hydrophilic bile acids synthesized by the rodent-specific enzymes Cyp2c70 and Cyp2a12. Mice with double deletions of Cyp2c70/Cyp2a12 (CYPDKO mice) have a human-like hydrophobic bile acid composition. PFIC-related gene mutations were induced in CYPDKO mice to determine whether these triple-gene-deficient mice are a better model for PFIC. To establish a PFIC3 mouse model using CYPDKO mice, we induced abcb4 gene deletion *in vivo* using adeno-associated viruses expressing SaCas9 under the control of a liver-specific promoter and abcb4-target gRNAs. Compared to Abcb4-deficient wild-type mice, Abcb4-deficient CYPDKO mice showed more pronounced liver injury along with an elevation of inflammatory and fibrotic markers. The proliferation of intrahepatic bile ductal cells and hematopoietic cell infiltration were also observed. CYPDKO/abcb4-deficient mice show a predominance of taurine-conjugated chenodeoxycholic acid and lithocholic acid in the liver. In addition, phospholipid levels in the gallbladder bile were barely detectable. Mice with both human-like bile acid composition and Abcb4-defect exhibit severe cholestatic liver injury and are useful for studying human cholestatic diseases and developing new treatments.

The bile is composed of water, bile acids, bilirubin, cholesterol, and fatty acids. Bile acids, the major components of bile, activate various signaling pathways by binding to specific nuclear receptors, such as the farnesoid X receptor (FXR) (1, 2). The disruption of the bile acid transport pathway in hepatocytes causes liver damage and inflammation, leading to liver fibrosis, cirrhosis, and hepatocellular carcinoma. Many hepatocyte transporters are involved in the influx and outflow of intracellular bile, and mutations or defects in these transporters and related genes can cause cholestasis and cholangitis including progressive familial intrahepatic cholestasis (PFIC) (3). In humans, PFIC occurs during childhood and requires a liver transplant. To date, at least six PFIC-related genes have been identified: *ATP8B1*, *ABCB11*, *ABCB4*, *TJP2*, *NR1H4*, and *MYO5B* (4). ABCB4, which belongs to the ATP-binding cassette transporter family, is located along the canaliculus of hepatocytes (5) and transports phosphatidylcholine into bile (6, 7). The bile fluid contains phospholipids and cholesterol, which play a role in decreasing the toxic detergent effects of bile. Therefore, ABCB4 deficiency results in cholangitis and cholestasis-induced liver injury. Mice lacking PFIC-related genes have been used to reproduce the symptoms of human PFIC in an experimental model (8, 9). Abcb4-deficient mice lack bile phospholipids and exhibit liver injury owing to increased bile toxicity. AST and ALT levels are elevated at 4–8 weeks of age and cirrhosis with fibrosis develops at 8–12 weeks. Abcb4 suppression also induces hepatocellular carcinoma formation in older mice (10). However, the symptoms in these mouse models are generally milder than those in human PFIC3, which progresses rapidly and severely and requires liver transplantation. This phenotypic difference may be attributable to the differences in bile acid composition between humans and mice (11). Most bile acids in mice are hydrophilic muricholic acids (MCAs) because mice express specific bile acid-metabolizing enzymes that are not found in humans: Cyp2c70 and Cyp2a12 (12). Mice, but not humans, have a metabolic pathway that converts hydrophobic bile acids to hydrophilic MCAs and cholic acid (CA) using Cyp2c70/Cyp2a12. Unlike wild-type mice, Cyp2c70/Cyp2a12 double-deficient mice (CYPDKO) lack MCA and have a bile acid composition similar to that of humans, containing more hydrophobic bile acids. We considered utilizing CYPDKO mice to reproduce human PFIC because these mice with human-like bile acids would manifest more severe liver injury than wild-type mice.

The liver maintains homeostasis and participates in the metabolism of drugs, lipids, cholesterol, and bile acids. Gene transgenic and knockout mice are useful for functional analyses of transcription-related factors, metabolic enzymes, and transporters that regulate these metabolic pathways. Genome-editing enzymes such as CRISPR/Cas9 are useful for the rapid establishment of gene knock-out and knock-in mice (13, 14, 15). An in vivo genome editing method using Cas9 transgenic mice has been reported (16). However, multiple steps and genetically modified mice are required to generate tissue-specific, genetically deficient mice. The introduction of the Cas9 protein using an adeno-associated virus (AAV) has been recently reported to enable direct genome editing in adults (17, 18). AAV allows easy in vivo gene transfer to various organs such as the liver (19, 20). For example, gene deletion and suppression mouse models have been established using Cas9-induced indel gene editing and the Cas9KRAB fusion protein in the liver (21). Studies on in vivo genome editing and gene therapy for liver diseases, such as hemophilia, have reported the use of an AAV-dependent Cas9 delivery system (22, 23, 24), suggesting that these AAV-dependent genome-editing systems are useful for liver-specific gene knockout methods in normal mice without the Cas9 transgene in the genome (25).

In this study, we generated PFIC model mice with a human bile acid-like hydrophobic composition and analyzed the species differences in bile acid composition in cholestasis models. The induction of a new gene mutation in CYPDKO mice requires extensive genetic engineering and mating. Therefore, we used a system that somatically induces gene deletion using AAV. Liver-specific gene-deficient mice were established within a short period by expressing SaCas9 under the control of a human antitrypsin (hAAT) promoter. We induced Abcb4-deficiency in CYPDKO and wild-type C57BL/6J (B6) mice to compare PFIC physiology with different hydrophobic and hydrophilic bile acid compositions. Abcb4 depletion in CYPDKO mice induced more severe liver damage and fibrosis within a shorter period than in wild-type mice. This is the first study to demonstrate the importance of species-specific differences in bile acid composition in an animal model of cholestasis. This PFIC mouse model is a useful tool for studying human PFIC and developing new treatments.

## Materials and Methods

### Experimental animals

C57BL6/J mice were purchased from Nihon SLC and CLEA Japan, Inc. Mutant green fluorescent protein (GFP) transgenic mice (#197) have also been established (26). Cyp2a12/2c70 double knockout (CYPDKO) mice have been described previously (12). Mice were maintained on a 12-h dark/light cycle with free access to food (CA-1) and water. Ursodeoxycholic acid (UDCA) water (2 mM), prepared by diluting UDCA (DS Pharma Animal Health Co), was administered as drinking water to CYPDKO mice and changed to normal water 1 week before AAV injection. In CYPDKO mice, the increased hydrophobicity of bile acids caused a decrease in reproductive ability, and 20%–25% of mice died after weaning. Feeding UDCA to CYPDKO mice not only ameliorated frequent miscarriages and unexpected deaths after weaning but also suppressed spontaneous liver injury (27). In addition, the discontinuation of UDCA returned the BA composition to the original human-like composition within two weeks (27). Since samples were collected 4–5 weeks after AAV administration, total 5–6 weeks had passed since UDCA administration was stopped. These mice were sacrificed between 12:00 and 16:00 under anesthesia with isoflurane after fasting for 4 h with free access to water. In these Abcb4-deficient studies, male CYPDKO and C57BL6/J mice (10–13 weeks old) were used for AAV injection because of spontaneous liver injury in female CYPDKO mice. The animal experimental protocols were approved by the Institutional Animal Care and Use Committee of Tokai University (approval numbers: 243,010, 232,006, 232,008, 221,011, 221,105, 211,068, 201,051, and 204,009).

### Virus construction

A liver-specific promoter-loaded version of the rAAV2-LSP1 vector (20) and the rAAV2 vector was used to introduce SaCas9 (Takara Bio Inc.). A rAAV2 vector containing the human-U6 promoter was used for gRNA expression. The gRNA sequences were designed using CRISPOR (http://crispor.tefor.net/). The target sequences of the gRNA are listed in supplemental Table S1. AAV was produced by co-transfecting HEK293T cells with the AAV8 capsid (Penn Vector Core, University of Pennsylvania) and helper plasmids (Takara Bio Inc.). HEK293T cells were cultured in Dulbecco’s modified Eagle’s medium (DMEM) supplemented with 10% fetal bovine serum and 1% penicillin-streptomycin-L-glutamine. After 16 h, the medium was replaced with DMEM supplemented with 2% fetal bovine serum and 1% penicillin-streptomycin-L-glutamine solution and cultured for 2 days, and all cells were collected. AAV was purified from the cells and concentrated using the AAVPro Purification Kit (Takara Bio Inc.). AAV titers were measured using the AAVpro Titration Kit Ver2 (Takara Bio, Inc.) for RT-PCR.

### AAV injection into mice for gene deletion

After anesthesia was induced through isoflurane inhalation via the oral cavity, AAVs expressing SaCas9 in combination with either EGFP-gRNA or three types of gRNAs for target genes were administered intraperitoneally (2.0–3.0 × 10^11^ vg/mouse). For Abcb4 deletion, 10-13-weeks-old CYPDKO and C57BL/6J male mice were used. Mice infected with AAVs expressing SaCas9 and EGFP-gRNA, or mice without AAVs were used as negative controls (NTC). To select mice for AAV injection of either EGFP control gRNA or target gene gRNAs, mice of approximately the same weight were randomly selected. One CYPDKO/Abcb4-deficient mouse was excluded from the analyses because of liver atrophy caused by unknown reasons.

### Statistical analysis

Student’s t-tests and one way ANOVA (for analysis of more than three groups) were performed using Prism7 (GraphPad Software), SDs were calculated, and statistically significant differences were determined.

All other methods are shown in the Supporting Materials and supplemental Tables S1 and S2.

## Results

### Liver-specific genome-editing using AAV and CRISPR/Cas9

A system for identifying genome editing efficiency by introducing gRNA around the mutation site in mutant GFP transgenic mice has been reported (26), in which Cas9 and gRNA introduction causes an indel mutation that corrects the reading frame of the gene with a certain probability and restores mutant-GFP activity. Therefore, we used this system to analyze the liver-specific promoter activity during AAV infection (supplemental Fig. S1A). A liver-specific AAV vector reportedly consists of an Apolipoprotein E enhancer, a hAAT promoter, and a WPRE sequence involved in RNA stabilization (20). Three SaCas9 expression vectors, with or without these components, were constructed, and the activity of each vector was evaluated (supplemental Fig. S1B). Mutant GFP-mice (9-week-old) were infected with AAV-a,-b, and c vectors and AAV expressing gRNA against mutant GFP (1.1 × 10^11^ vg/mouse), and livers were analyzed with a fluorescence microscope after 4 weeks of infection. When mutant-GFP adult mice were infected with these liver-specific SaCas9-expressing AAVs and human U6 promoter gRNA-expressing AAVs, a similar recovery of GFP fluorescence was observed for all three expression vectors, suggesting that the hAAT promoter was sufficient for hepatic Cas9 expression (supplemental Fig. S1C). We also analyzed whether genome editing by the hAAT-promoter AAV was possible in juvenile mice. Mutant GFP-mice (2-week-old) were transfected with AAV-c and AAV expressing gRNA against mutant GFP (0.7–1.2 × 10^11^ vg/mouse), and livers and other organs were analyzed with a fluorescence microscope after 2–8 weeks. Significant GFP fluorescence was observed in the liver, whereas no recovery of luminescence was observed in other organs (supplemental Fig. S1D, E). Therefore, we found that a short hAAT promoter expressing SaCas9 AAV is sufficient as an expression control region to enable liver-specific genome editing.

### Effect of Abcb4 deficiency on human and mouse bile acid composition

**Fig. 1:**
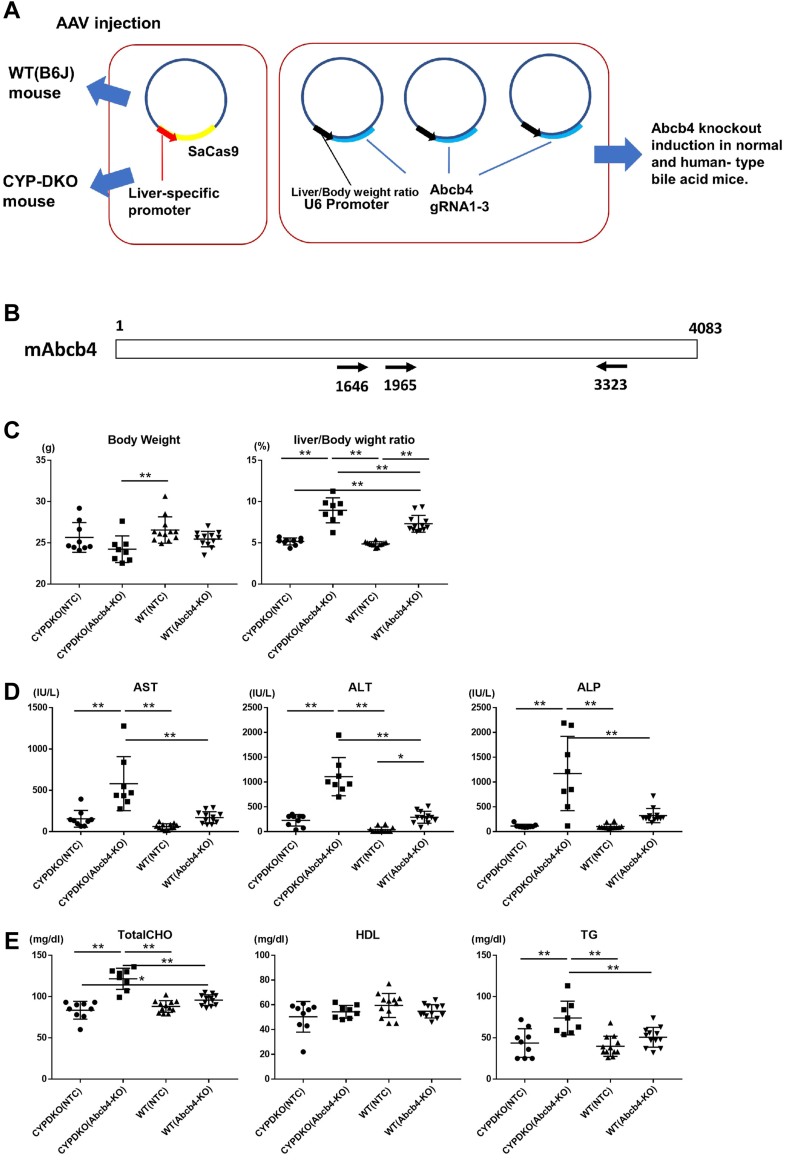
Effect of Abcb4 deficiency in human bile acid-model by AAV-triple CRISPR method. A: Generation of liver-specific Abcb4-deletion mice by the triple CRISPR method. B: Three gRNAs against the Abcb4 gene. AAVs expressing gRNAs targeting the indicated sites in the CDS region were generated. C: Changes in body weight and liver/body weight ratio. D: Changes in liver-injury markers serum levels. E: Changes in serum total cholesterol (TotalCHO), HDL, and triglycerides (TG). (n = 9 for CYPDKO/NTC, n = 8 for CYPDKO/Abcb4-KO, n = 12 for WT/NTC, and n = 12 for WT/Abcb4-KO). Results are represented as mean ± SD (one-way ANOVA, ∗*P* < 0.05, ∗∗*P* < 0.01).

As shown earlier, the combination of the hATT promoter and SaCas9 efficiently induced liver-specific gene deletions. Therefore, using AAV, we introduced SaCas9 and Abcb4-gRNA into CYPDKO mice, which are human bile acid-like model mice, and wild-type C57BL/6J mice (control) to induce Abcb4 deletion (Fig. 1A, B). Co-introduction of the liver-specific promoter SaCas9 and the three gRNAs for Abcb4 using AAVs efficiently induced target gene deletion. We compared the relationship between changes in bile acid composition and Abcb4-deficiency in both male wild-type and CYPDKO mice. Almost 4–5 weeks after the introduction of AAV, the liver, other tissues, and serum were collected and analyzed. Abcb4 deficiency did not affect body weight. The liver/body weight ratio increased in both CYPDKO and wild-type mice with Abcb4 deletion (Fig. 1C). Similar to previous results (28), serum levels of liver injury markers (AST, ALT, and ALP) also tended to be elevated following Abcb4 deletion in the wild-type mice, whereas the upregulation of injury markers in WT/Abcb4-deficient mice was slight because of short-term gene deletion (4–5 weeks after AAV injection) compared to previous gene deletion studies. In contrast, liver injury markers were significantly increased in CYPDKO/Abcb4-deficient mice, indicating that more severe liver injury was induced by the combination of Abcb4 deletion and human-like bile acid composition, even within a short period (Fig. 1D). In addition, a slight increase in total cholesterol and triglyceride serum levels was observed in the CYPDKO/Abcb4-deficient mice (Fig. 1E).

**Fig. 2:**
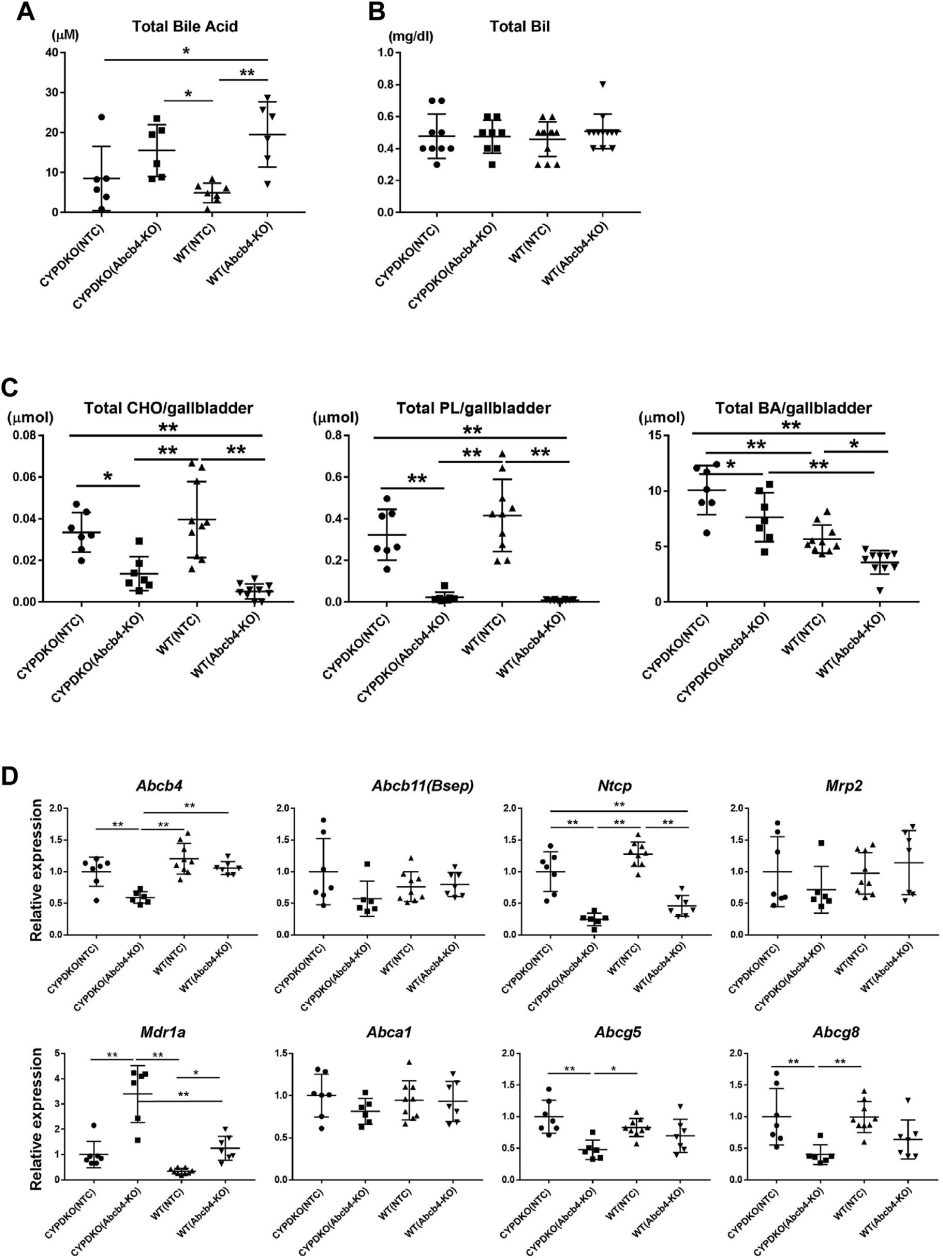
Bile acid and cholesterol levels in the serum and gallbladder derived from CYPDKO/Abcb4-deficient mice. A: changes in serum total bile acid levels (n = 6 for CYPDKO/NTC, n = 6 for CYPDKO/Abcb4-KO, n = 7 for WT/NTC, and n = 6 for WT/Abcb4-KO). B: Changes in serum total bilirubin (Bil) levels (n = 9 for CYPDKO/NTC, n = 8 for CYPDKO/Abcb4-KO, n = 12 for WT/NTC, and n = 12 for WT/Abcb4-KO). C: Changes in cholesterol (CHO), phospholipid (PL), and total bile acid (BA) levels derived from the gallbladder bile (n = 7 for CYPDKO/NTC, n = 7 for CYPDKO/Abcb4-KO, n = 10 for WT/NTC, and n = 10 for WT/Abcb4-KO). D: Gene expression changes of bile acid and cholesterol metabolism-related genes in the liver. The expression of genes in CYPDKO/NTC mouse livers was set to 1.0 (n = 7 for CYPDKO/NTC, n = 6 for CYPDKO/Abcb4-KO, n = 9 for WT/NTC, and n = 7 for WT/Abcb4-KO). Results are represented as mean ± SD (one-way ANOVA, ∗*P* < 0.05, ∗∗*P* < 0.01.).

Next, total bile acid and bilirubin serum levels were measured. Both Abcb4-deficient mice were found to have elevated total bile acid serum levels, indicating that Abcb4 deletion induced cholestasis within a short period in both CYPDKO and wild-type mice (Fig. 2A). In contrast, serum total bilirubin (Bil) levels did not increase (Fig. 2B).

Bile is composed mainly of bile acids, cholesterol, and phospholipids. Quantitative analysis of these components showed that the amount of total bile acids in the gallbladder bile was higher in CYPDKO mice than in wild-type mice (Fig. 2C). Furthermore, in Abcb4 gRNA-treated mice, almost no phospholipids were detected in the bile, indicating that Abcb4-genome editing hepatocytes had defective phospholipid transporters. Furthermore, cholesterol levels were reduced owing to Abcb4 deficiency. These results indicate that the bile of CYPDKO/Abcb4-deficient mice had reduced cholesterol and phospholipid levels and a high bile acid composition. Thus, the expression of bile and cholesterol transporters in hepatocytes was analyzed (Fig. 2D). There was almost no change in Abcb4 mRNA expression in Abcb4-deficient wild-type mice. In contrast, the amount of mRNA was almost halved down-regulated in Abcb4-deficient CYPDKO-mice. Since a certain level of mRNA expression was observed in both Abcb4-deficient mice, it was suggested that mutant or shortened Abcb4 mRNAs existed in this genome editing model despite the functional loss of Abcb4 such as the absence of phospholipids in the gallbladder bile (Fig. 2C, D). In addition, changes in the expression of Abcb11, Mrp2, and Abca1 were barely observed following Abcb4 deletion. The expression of Ntcp, which is involved in the uptake of bile acids from the small intestine into hepatocytes, was decreased by Abcb4 deficiency, indicating changes in the bile enterohepatic circulatory system. Furthermore, the expression of Abcg5 and Abcg8, which are involved in cholesterol excretion from hepatocytes to bile, was decreased by Abcb4 deficiency in CYP/DKO mice, consistent with the decrease in cholesterol levels in the gallbladder bile. In contrast, Mdr1a expression increased in CYPDKO/Abcb4-deficient mice, which may be a response to severe liver injury.

### Abcb4 deficiency with hydrophobic bile acid composition induced liver inflammation, bile duct hyperplasia, and fibrosis

**Fig. 3:**
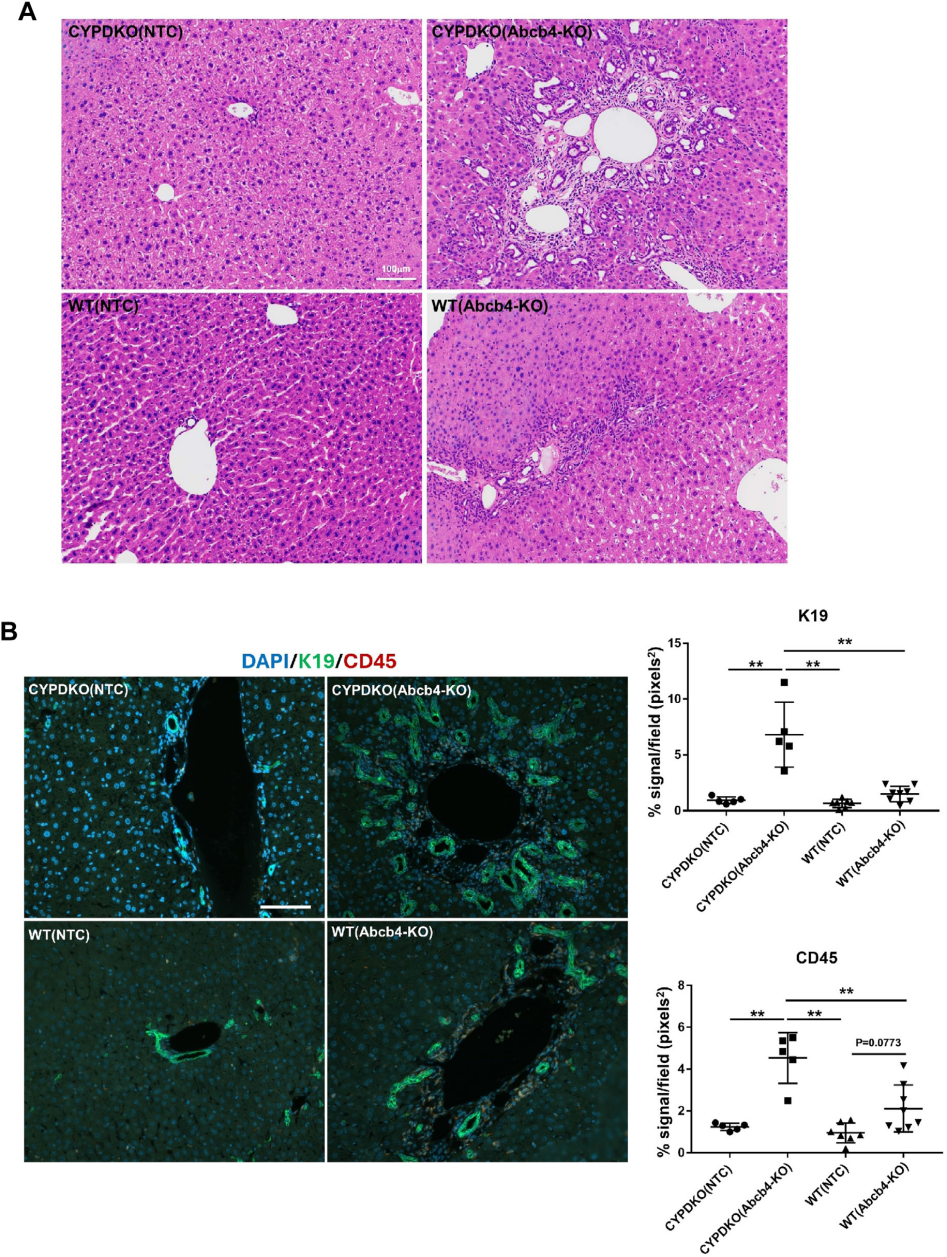
Morphological changes in CYPDKO/Abcb4-deficient mouse liver. A and B: induction of blood infiltration and bile duct hyperplasia by Abcb4 deficiency. Hematoxylin and Eosin staining (A) and K19 and CD45 immunostaining (B) were performed. B (right panels): K19 and CD45 stains quantified the amount of biliary cell proliferation and hematopoietic cell infusion using the ImageJ software (n = 5 for CYPDKO/NTC mouse livers, n = 5 for CYPDKO/Abcb4-KO mouse livers, n = 7 for WT/NTC mouse livers, and n = 8 for WT/Abcb4-KO mouse livers). White line, 100 μm. Results are represented as mean ± SD (one-way ANOVA, ∗*P* < 0.05, ∗∗*P* < 0.01.).

**Fig. 4:**
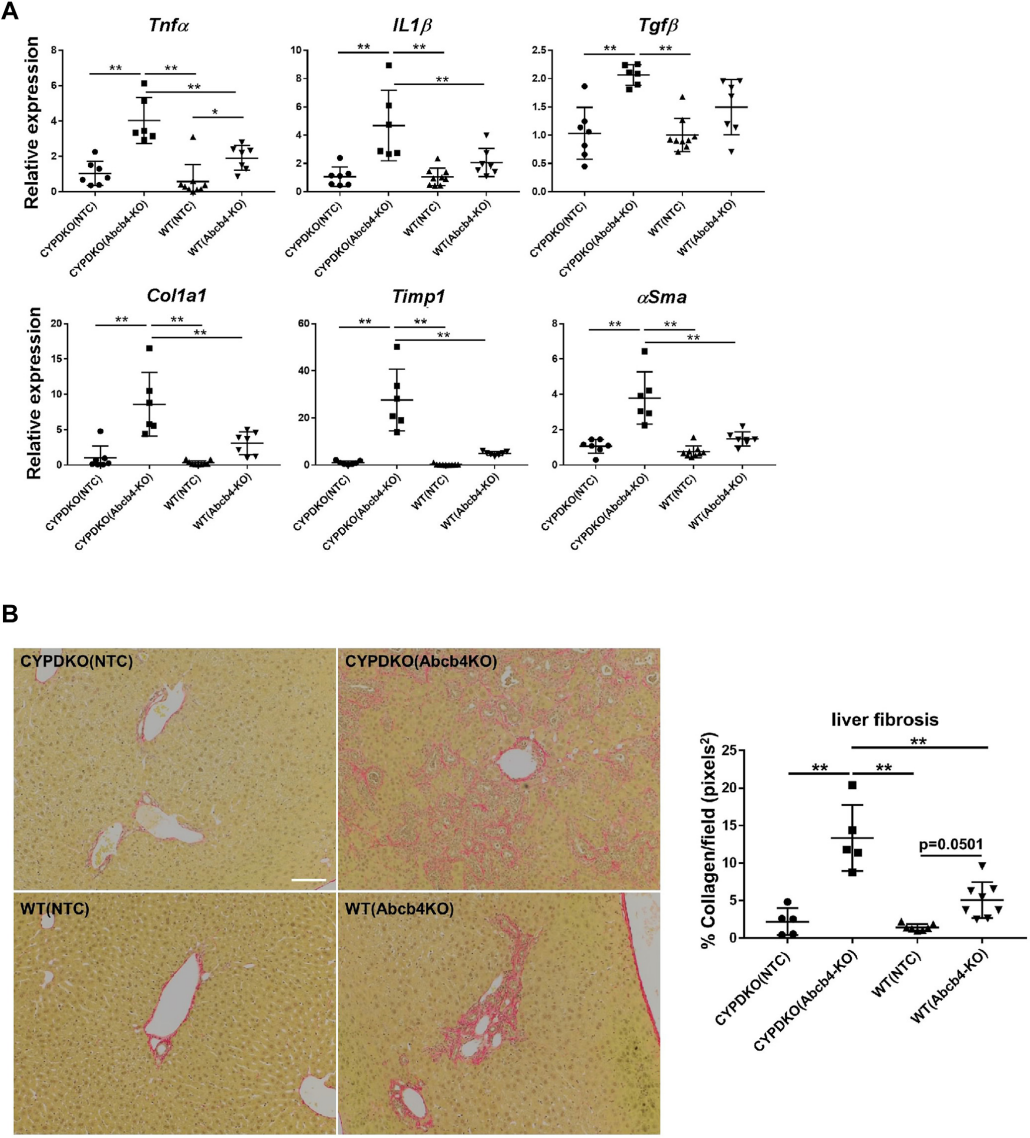
Inflammatory and fibrosis in CYPDKO/Abcb4-deficient mice. A: gene expression changes of inflammatory and fibrotic markers in the liver. The expression of genes in CYPDKO/NTC mouse livers was set to 1.0 (n = 7 for CYPDKO/NTC, n = 6 for CYPDKO/Abcb4-KO, n = 9 for WT/NTC, and n = 7 for WT/Abcb4-KO). (B) Analysis of intrahepatic fibrosis with Sirius red staining. B (right panel): Sirius red stain was performed to visualize the liver fibers and quantify the amount of liver fibers using the ImageJ software (n = 5 for CYPDKO/NTC mouse livers, n = 5 for CYPDKO/Abcb4-KO mouse livers, n = 7 for WT/NTC mouse livers, and n = 8 for WT/Abcb4-KO mouse livers). White line, 100 μm. Results are represented as mean ± SD (one-way ANOVA, ∗*P* < 0.05, ∗∗*P* < 0.01.).

We analyzed bile ductal cell proliferation and hematopoietic cell infusion induced by Abcb4 deletion. In a normal genetic background, Abcb4 deletion non-significantly increased the proliferation of K19-positive bile ductal cells around the portal veins (Fig. 3A, B, WT/Abcb4-KO). Human-like bile acid composition in CYPDKO mice (CYPDKO/NTC) alone does not cause bile duct hyperplasia. In contrast, the combination of Abcb4 deletion and human-like bile acid composition (CYPDKO/Abb4-KO) resulted in periportal CD45-positive hematopoietic cell infiltration, consistent with the exacerbation of liver injury as shown by serum marker levels, leading to significantly increased K19-positive bile ductal cell proliferation (Fig. 3B, right panels).

We analyzed changes in gene expression in the liver following Abcb4 deletion. The expression of inflammatory cytokines (TNF-α and IL-1β) and fibrosis-related TGF-β was increased when Abcb4-gene deletion was induced in both CYPDKO and wild-type mice (Fig. 4A). Collagen 1a1 and TIMP1 were significantly upregulated in CYP/DKOAbcb4-deficient mice, suggesting the importance of hydrophobic bile acid composition in liver fibrosis. In addition, smooth muscle actin (αSMA), an activated stellate cell marker, was induced by Abcb4 deletion. Next, liver fibrosis caused by collagen fiber deposition was analyzed because the expression of liver fibrosis marker genes was upregulated by Abcb4-deletion in CYPDKO mice. As shown in the quantitative results, a slight deposition of collagen fibers was observed around the periportal bile duct structure in mice with Abcb4 deficiency alone. In CYPDKO/Abcb4 mice, strong fibrosis was observed around the hyperplastic bile ducts within a relatively short period (almost 4–5 weeks) of Abcb4 deficiency (Fig. 4B). Thus, Abcb4 deficiency in a human bile acid-like composition may induce stronger liver injury and fibrosis than that in mice with a hydrophilic bile acid composition.

### Changes in intrahepatic bile acid and lipid composition caused by Abcb4 deficiency

**Fig. 5:**
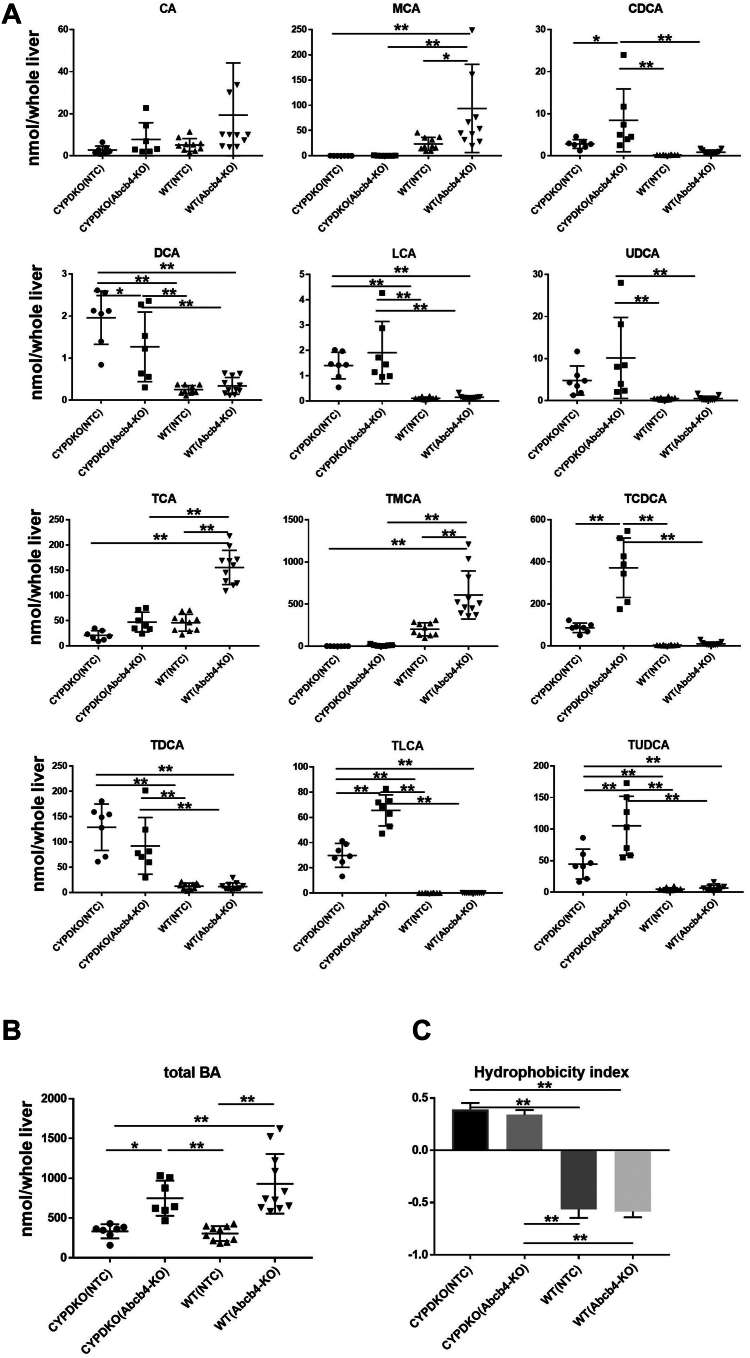
Bile acid composition in the liver of CYPDKO/Abcb4-deficient mice. A and B: Level changes of various and total bile acids in the whole liver derived from WT and CYPDKO mice with Abcb4 deficiency. C: the hydrophobicity indices of total bile acid in the liver. Hydrophobicity index was calculated as a percentage-weighted mean of individual bile acid hydrophobicity as shown in supplemental Table S3 (n = 7 for CYPDKO/NTC mouse livers, n = 7 for CYPDKO/Abcb4-KO mouse livers, n = 10 for WT/NTC mouse livers, and n = 11 for WT/Abcb4-KO mouse livers). Results are represented as mean ± SD (one-way ANOVA, ∗*P* < 0.05, ∗∗*P* < 0.01.).

Abcb4 deficiency alters bile acid composition in the liver. We analyzed whether the bile acid composition in the whole liver was altered by Abcb4 deletion in wild-type and CYPDKO mice using Liquid Chromatography-Mass Spectrometry (Fig. 5A and supplemental Table S3). In wild-type mice, most bile acids are taurine-conjugated, as previously reported (12), and taurine-conjugated MCAs are abundant. Abcb4 deficiency resulted in increased levels of taurine-conjugated CA and MCA in the wild-type genetic background. In contrast, free and taurine-conjugated MCAs were hardly detected in the livers of CYPDKO mice, whereas other taurine-conjugated bile acids such as chenodeoxycholic acid (TCDCA), deoxycholic acid (TDCA), lithocholic acid (TLCA), and TUDCA were elevated. In the CYPDKO genetic background, free and taurine-conjugated CDCA and LCA levels were increased following Abcb4 deletion. The ratio of bile acid composition revealed that wild-type mice mainly had MCA, TCA, and TMCA in the liver (supplemental Fig. S2). In contrast, CYPDKO mice had few MCA and TMCA. CYPDKO/NTC mice mainly had TDCA (almost 40%), whereas CYPDKO/Abcb4 mice mainly had TCDCA (almost 45%).

Total bile acid levels in the whole liver remained unchanged in CYPDKO/NTC mice. In contrast, total liver bile acid levels were significantly increased by Abcb4 deletion in both wild-type and CYPDKO mice, suggesting that liver cholestasis occurred due to Abcb4 deletion (Fig. 5B). We then calculated the hydrophobicity index (HI) of the bile acid composition in the liver. Wild-type mice, which contain more hydrophilic bile acids such as MCA, showed a low HI regardless of the presence or absence of Abcb4. In contrast, CYPDKO mice, which lacked the MCA, showed a high HI (Fig. 5C). Therefore, it is thought that the degree of liver damage is suppressed, even in WT/Abcb4-deficient mice, which have a high amount of intrahepatic bile acids.

**Fig. 6:**
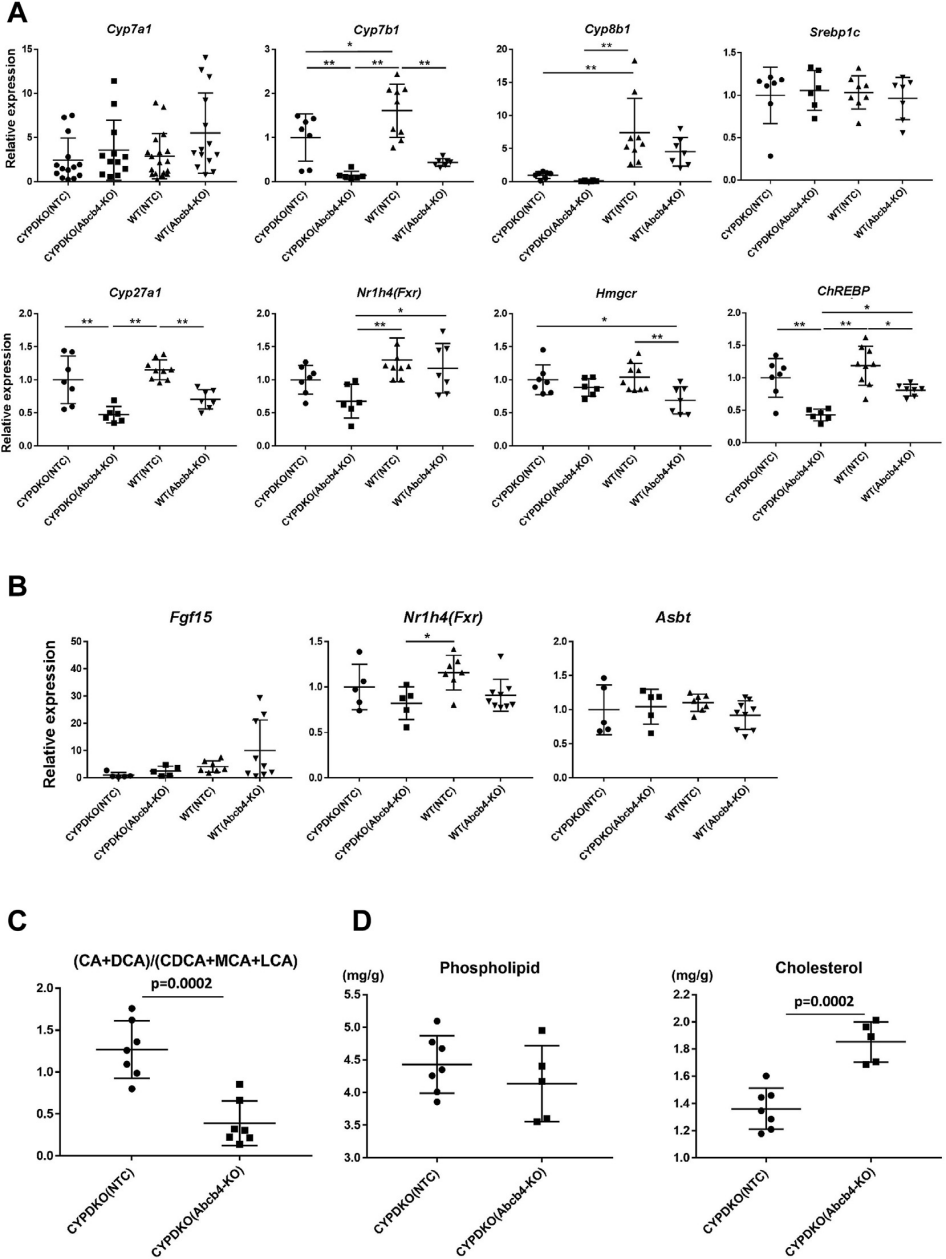
Changes of bile acid metabolic pathways in the Abcb4-deleted CYPDKO mice. A: Expression of bile acid and lipid synthesis-related enzymes and transcription factors in the liver. The expression of genes in CYPDKO/NTC mouse livers was set to 1.0 (n = 7 for CYPDKO/NTC, n = 6 for CYPDKO/Abcb4-KO, n = 9 for WT/NTC, and n = 7 for WT/Abcb4-KO). B: Gene expression changes of bile acid metabolism-related genes in the terminal ileum. The expression of genes in CYPDKO/NTC mouse terminal ileum was set to 1.0 (n = 5 for CYPDKO/NTC, n = 5 for CYPDKO/Abcb4-KO, n = 7 for WT/NTC, and n = 9 for WT/Abcb4-KO). C: (CA + DCA)/(CDCA + MCAs + LCA) ratio of bile acids in the liver derived from the Abcb4-deleted CYPDKO mice (n = 7 for CYPDKO/NTC mouse livers, and n = 7 for CYPDKO/Abcb4-KO mouse livers). D: Phospholipid and cholesterol in the CYPDKO and CYPDKO/Abcb4-KO mouse livers. After extraction of lipids from mice livers, the amount of phospholipid and cholesterol were analyzed (n = 7 for CYPDKO/NTC mouse livers and n = 5 for CYPDKO/Abcb4-KO mouse livers). Results are represented as mean ± SD (A, B, One way ANOVA; C, D, Student’s *t* test, ∗*P* < 0.05, ∗∗*P* < 0.01.).

Bile acid synthesis is regulated by several CYP family genes. Analysis of bile acid synthase gene expression in the liver revealed that Cyp7b1 and Cyp27a1 were downregulated by Abcb4 deletion in both wild-type and CYPDKO mice (Fig. 6A). CYPDKO-induced human bile acid-like changes decrease Cyp8b1 expression. In particular, Cyb8b1 expression was barely detectable in CYPDKO/Abcb4-deficient mice. Abcb4-deletion does not significantly suppress Cyp7a1 expression in wild-type or CYPDKO mice. Expression of Nr1h4 (Fxr) was downregulated by Abcb4 deletion in CYPDKO mouse livers. In contrast, the expression of Hmgcr, a cholesterol synthesis enzyme, was downregulated in WT/Abcb4-deficient mice. Expression of lipid metabolism-related transcription factors ChREBP but not Srebp1c was downregulated by Abcb4-deletion in both WT and CYPDKO mice. In the small intestine, Nr1h4 and fibroblast growth factor (FGF) 15 are involved in bile acid metabolism. In mice, expression of Cyp7a1 is mainly regulated by the intestinal Fgf15 pathway via Nr1h4 (29). We analyzed the expression of these genes in the terminal ileum; however, no significant changes were observed in Fgf15 expression, although a slight decrease of Nr1h4 expression was observed in CYPDKO/Abcb4-deficient mice (Fig. 6B).

As shown above, in wild-type mice livers, more than half of the bile acid composition was of hydrophilic taurine-conjugated MCAs regardless of the presence or absence of Abcb4 deficiency. In contrast, TDCA was the major bile acid in CYPDKO mice, and TCDCA was the major bile acid in CYPDKO/Abcb4-deficient mice (Fig. 5A). In the bile acid metabolic pathways, Cyp8b1 regulates the ratio of primary bile acid CA and secondary bile acid DCA (CA + DCA) to primary bile acids CDCA, αMCA, and βMCA and secondary bile acids LCA and ωMCA (CDCA + MCAs + LCA). Cyp27a1 and Cyp7b1 also regulate the production of (CDCA + MCAs + LCA). Thus, the (CA + DCA)/(CDCA + MCA + LCA) ratio indicates which synthetic pathways and Cyp enzymes are important for bile acid composition. The (CA + DCA)/(CDCA + MCA s + LCA) ratio of wild-type mouse livers with or without Abcb4 deletion was low because of the high concentration of MCA. In contrast, there were very few MCAs in the livers of CYPDKO mice. Therefore, we compared the (CA + DCA)/(CDCA + MCA + LCA) ratio between CYPDKO/NTC and CYPDKO/Abcb4-deficient mice. This ratio was significantly decreased by the combination of CYPDKO and Abcb4-deletion (Fig. 6C). The synthesis or import of first bile acid CA and second bile acid DCA into the liver might be suppressed by the Abcb4-defect.

Abcb4 is a transporter that supplies phospholipids from hepatocytes to bile, and cholesterol is partially metabolized by bile acids. As shown in Fig. 2, phospholipid and cholesterol levels in the gallbladder bile were significantly downregulated by the deletion of Abcb4. We analyzed the hepatic phospholipid and cholesterol levels in CYPDKO/Abcb4-deficient mice. Phospholipid levels in the liver remained constant despite Abcb4 deficiency in CYPDKO mice, suggesting that cell membrane phospholipids, the main components of phospholipids in hepatocytes, are barely altered by the deletion of Abcb4. Abcb4 deletion increased cholesterol levels in the liver (Fig. 6D). The metabolism of cholesterol to bile acids may be inhibited by decreased expression of several CYP genes involved in bile acid synthesis, as observed in CYPDKO/Abcb4-deficient mice.

## Discussion

The Cas9 gene and gRNA for the target genes were expressed in vivo using AAVs, making them targets for disease reproducibility and treatment via genome editing (22, 30). In addition, the use of tissue-specific promoters for Cas9 protein expression by AAV enables the rapid induction of tissue-specific gene deletion (25). Here, we used the hAAT promoter as a liver-specific promoter for SaCas9 gene expression. When we analyzed this promoter activity using a genome-editing evaluation system in GFP-mutant mice, it was found to be highly specific, as it did not induce genome-editing in organs other than the liver. The hAAT promoter is very small and suitable while using AAV for the transduction of several genes other than Cas9. A method for introducing gRNA into Cas9-transgenic mice has also been reported as an in vivo gene modification system (16, 31) and these mice were recently used for cholestasis-related gene assays (32). Although this method has the advantage of producing and using less number of AAV, mice strains susceptible to gene modification are limited. Using Cas9-transgenic mice, inducing additional mutations in already established gene-deficient mice is laborious because Cas9-transgenic mice must be mated with target gene-deficient mice. Therefore, developing an enhanced system that efficiently introduces both Cas9 and gRNA in a tissue-specific manner to induce genome editing would be advantageous for analyzing existing gene-knockout mice.

The hydrophobicity of bile acids contributes to the induction of liver injury in cholestasis. Hydrophobic bile acid composition induced by the CA diet in hepatic reductase-null mice is involved in ATP8b1-and Abcb4-deletion-induced liver injury (11). In contrast, the addition of hydrophilic tetrahydroxylated bile acid improves cholestatic liver injury in Abcb4-knockout mice (28, 33). The major difference in bile acid composition between mice and humans is the high level of hydrophilic MCAs in murine bile, owing to the expression of mouse-specific bile acid-metabolizing enzymes and the abundance of taurine-conjugated bile acids. A human bile acid-like mouse model genetically deleted for the enzyme involved in MCA synthesis was used for these analyses because the toxicity of bile acid is similar to that of human synthesis (12). For example, when CYPDKO mice were treated with UDCA, a therapeutic drug for liver damage, the hydrophobicity of bile acids was reduced, and the liver damage caused by CYPDKO was suppressed in male mice. In contrast, the addition of UDCA increased intrahepatic LCA in female mice, leading to increased liver injury (27). In autoimmune diseases, such as primary biliary cholangitis, human-like changes in bile acid composition caused by CYPDKO affect hepatotoxicity and induce pathological changes (34). Thus, mice with bile acid compositions similar to those of humans are thought to be extremely useful for analyzing liver damage, such as cholestasis.

In this study, the PFIC3-related gene, Abcb4, was analyzed to examine whether human-like bile acid composition alters PFIC physiology in mouse models. We used an AAV-mediated genome-editing system to induce Abcb4-deletion in CYPDKO mice. Wild-type mice with an Abcb4-deletion have been reported, and increased serum levels of liver injury markers and associated changes in liver tissue (e.g., hyperplasia of bile ducts) have been observed several weeks after birth (28). Abcb4 is a phospholipid transporter that is transported to the bile. It is possible that Abcb4 deletion increases bile toxicity by decreasing phospholipid concentrations in bile. We found that phospholipid and cholesterol levels in gallbladder bile were significantly down-regulated by the deletion of Abcb4 in both wild-type and CYPDKO mice. In addition, total bile acid levels in the serum and liver increased in both WT/Abcb4-deficient and CYPDKO/Abcb4-deficient mice. However, Abcb4-deletion induces more severe liver injury in CYPDKO mice than in wild-type mice. Bile acids in the livers of wild-type mice were predominantly composed of taurine-conjugated CA and taurine-conjugated MCAs. Hydrophobicity indices of total bile acids in the liver revealed a significant difference in hydrophobicity and toxicity between the bile acid compositions of CYPDKO and wild-type mice. Abcb4 deletion in wild-type mice did not induce severe liver injury after AAV infection in this short-term study (4–5 weeks). In contrast, Abcb4 deletion induced severe liver injury, bile ductal cell hyperplasia, and fibrosis in CYPDKO mice, which contain more toxic hydrophobic bile acids. These results indicate that the unusual regulation induced by Abcb4 deletion with humanized hydrophobic bile acid composition may be involved in hepatic injury, apoptosis, and inflammation. As shown in the previous Abcb4 KO mice reports (9), serum bilirubin was not significantly upregulated. Our study revealed that serum bilirubin levels were not altered by Abcb4 deletion in the humanized hydrophobic bile acid composition, which induced liver injury and fibrosis. The reason remains unknown but might be due to differences in bilirubin metabolism between humans and mice.

The molecular mechanism by which Abcb4 deletion induces liver injury remains unclear. Double-knockout mice with Abcb4 and either the TRAIL receptor or TLR4 revealed that Abcb4-deleted liver injury is involved in apoptosis- and inflammation-related mechanisms (35, 36). In addition, several cholesterol and bile acid metabolic pathways were changed by the deletion of Abcb4. When Abcb4 was deleted in CYPDKO mice, the amounts of CDCA, LCA, and UDCA increased in CYPDKO/Abcb4-deficient mice, the molecular mechanism underlying the increase in the amount of these bile acids is unknown. Bile acid synthesis is divided into the classic pathway and the alternative pathways (37). Cyp7a1 and Cyp8b1 mainly regulate the classic pathway and Cyp27a1 and Cyp7b1 mainly regulate the alternative pathway. Particularly, Cyp8b1 determines the balance of CA and CDCA through the classic pathway. In this study, CYP8b1 was significantly suppressed in both CYPDKO/NTC- and CYPDKO/Abcb4-deficient mice, suggesting that a different mechanism is responsible for the alteration of bile acid composition in Abcb4 deficiency. In addition, secondary bile acids, such as DCA, LCA, and UDCA, are metabolized in the intestine. These metabolic interactions in both the liver and intestine may have caused the formation of a specific bile acid composition and contributed to severe liver injury in CYPDKO/Abcb4-deficient mice.

In a conventional PFIC3 mouse model, the abcb4 gene deletion was induced in wild-type mice, which have a high content of hydrophilic bile acids, resulting in a longer time required for the induction of liver damage and fibrosis. In this study, using CYPDKO mice with a human-like bile acid composition, we established a PFIC3 mouse model that more closely resembles the human pathology. In addition to PFIC, bile acid-related diseases, including primary biliary cholangitis, may be affected by differences in bile acid composition in analyses using a mouse model. Methods for introducing new mutations into existing CYPDKO mice include genome-editing of fertilized eggs from CYPDKO mice or mating existing Abcb4-deficient and CYPDKO mice. However, these methods require a considerable amount of time to establish the desired mouse strain. Using a method in which SaCas9 and target gRNAs were introduced using AAV to induce gene deletion via genome-editing in vivo, we detected phenotypic differences in Abcb4 deletions between wild-type and CYPDKO mice. The combination of human bile acid-like CYPDKO mice and the rapid genome-editing method using AAV demonstrated in this study may be useful for the rapid analysis of various bile acid-related diseases in mouse models. In addition, elucidation and development of treatment methods are expected to be the result of studies using mouse models.

## Data availability

Data are available from the corresponding author upon reasonable request.

## Supplemental data

This article contains supplemental data.

## Conflicts of interest

The authors declare that they have no known competing financial interests or personal relationships that could have appeared to influence the work reported in this paper:

The author, Akira Honda, is an Editorial Board Member for *Journal of Lipid Research* and was not involved in the editorial review or the decision to publish this article.
