# Supplementary material for: Abcb4-defect cholangitis mouse model with hydrophobic bile acid composition by in vivo liver-specific gene deletion
Source: J Lipid Res. 2024 Aug 5;65(9):100616. doi: 10.1016/j.jlr.2024.100616 (PMC11407928; doi:10.1016/j.jlr.2024.100616)
Supplement: Supplemental data [file mmc1.pdf]

Supporting Materials

Abcb4-defect cholangitis mouse model with hydrophobic bile acid composition by *in vivo* liver-specific gene deletion

Kota Tsuruya, Keiko Yokoyama, Yusuke Mishima, Kinuyo Ida, Takuma Araki, Satsuki Ieda, Masato Ohtsuka, Yutaka Inagaki, Akira Honda, Tatehiro Kagawa, and Akihide Kamiya

Supporting Materials and Methods

Analyses of AAV-induced genome mutation using mutant GFP mice

An approximately 2-week-old and 9-week-old mutant GFP-transgenic mouse (#197) was administered AAV-expressing SaCas9 and mutant GFP-targeting gRNA (Table S1). After the AAV injection, the livers were perfusion-fixed with 4% paraformaldehyde. Frozen sections were prepared using a cryostat and GFP fluorescence was observed using an Axio Imager M2 (ZEISS, Oberkochen, Germany).

Gene expression analyses using quantitative RT-PCR

Immediately after collection, the liver tissues were soaked in RNA solution (Thermo Fisher Scientific, Waltham, MA, USA), stored overnight at 4 °C, placed in RNAiso Plus (Takara Bio Inc.) along with stainless beads, and homogenized. Chloroform was added and the solution was centrifuged, after which the supernatant was separated, and RNA was extracted by ethanol precipitation. First-strand cDNA for quantitative PCR was synthesized from 0.5 μg of RNA using the ReverTra Ace qPCR RT Master Mix with gDNA Remover (TOYOBO, Osaka, Japan). The target gene expression was corrected for TATA-binding protein. Quantitative analysis of the target mRNA was performed using a universal probe library system (Roche Diagnostics, Basel, Switzerland), the TAMRA-FAM probe purchased from Takara Bio Inc., or SYBR PCR (Table S2).

Serum physiological marker analyses

After anesthesia, blood was collected from the hearts of the mice. Serum was separated using Bloodsepar (ImmunoBiological Laboratories Co., Ltd., Gunma, Japan). The levels of total cholesterol, HDL, triglycerides, AST, ALT, ALP, and total bilirubin were measured using Spotchem (Arkray. Inc, Kyoto, Japan). The ranges of measurements for each compound were as follows: total cholesterol 50–400 mg/dL; HDL, 10–150 mg/dL; triglycerides: 25–500 mg/dL; AST and ALT, 10–1000 IU/L; ALP, 38–113 IU/L; total bilirubin, 0.4‐1.5 mg/dL. Values lower than the detection limit were defined as the lowest values. Serum total bile acid levels were analyzed using Total Bile Acid Assay Kit (CBL-STA631, Cell Biolabs. Inc. San diego, CA) according to the product manuscript.

Histological analysis

The livers were fixed in 4% paraformaldehyde (FUJIFILM Wako Pure Chemical) overnight and embedded in paraffin. Paraffin-embedded sections were analyzed by hematoxylin and eosin and Sirius red staining using standard protocols (1). After each staining, pathological specimens were observed, and images were captured using a BX63 microscope (Olympus, Tokyo, Japan). The degree of fibrosis was calculated using image sections stained with Sirius Red. Five representative images were collected from each mouse liver sections and the positive signals were quantified using Image J software (Bethesda, MA).

Immunohistochemical staining was performed to detect keratin 19 (K19) and CD45 in bile ductal and hematopoietic cells. Paraffin-embedded sections were heated at 110 °C for 10 min in target retrieval solution (Dako, Santa Clara, CA, USA). After blocking with 5% donkey serum/ PBS, the sections were incubated with either rat anti-CD45 (550539, BD Biosciences, Franklin Lakes, NJ, USA) or rabbit anti-K19 (provided by Prof. Miyajima, University of Tokyo) antibodies overnight at 4 °C. After washing, the sections were incubated with DyLight594 anti-rat IgG (Thermo Fisher Scientific) and Alexa488 anti-rabbit IgG (Thermo Fisher Scientific) for 60 min at room temperature. The fluorescence was observed using an Axio Imager M2 microscope. The expression of K19 and

CD45 was calculated using image sections. Five representative images were collected from each mouse liver sections and the positive signals were quantified using Image J software.

Analyses of cholesterol, phospholipid and bile acid levels in the gallbladder

Mouse liver gallbladders were collected and biliary cholesterol and phospholipid concentration were determined using LabAssay Kits (FUJIFILM Wako Pure Chemical). Biliary total bile acid levels were analyzed using Total Bile Acid Assay Kit (CBL-STA631, Cell Biolabs. Inc.).

Analyses of bile acid composition in the liver

Liver samples were solubilized in 1M NaOH/water at 80 °C for 20 min; After the addition of internal standards and 0.5 mol/L potassium phosphate buffer (pH 7.4), bile acids were extracted and quantified by Liquid Chromatography-Mass Spectrometry. The mass analysis protocol has been previously described (2). The hydrophobicity indices were calculated using the individual bile acid data from the previous report (3). The hydrophobicity indices of individual bile acids not listed in the previous reports were obtained from our HPLC data and tentatively defined as ωMCA (-0.69), αMCA (-0.68), βMCA (-0.62), TωMCA (-0.85) and LCA (1.22).

Liver lipid purification and analyses.

Liver lipids (CYPDKO/NTC, n=7; CYPDKO/Abcb4-KO, n=5) were purified as previously described (4). The frozen liver tissues were weighed and homogenized in distilled water. Lipids were extracted from the homogenate with three volumes of 2:1 (v/v) chloroform and methanol. After centrifugation, the lower layers were collected and evaporated. The precipitated lipids were dissolved in isopropyl alcohol-Triton X-100 (9:1 v/v). The phospholipids and cholesterol in the extracts were analyzed using LabAssay Kits (FUJIFILM Wako Pure Chemical).

References

1. Lopez-De Leon A, and Rojkind M. 1985. A simple micromethod for collagen and total protein determination in formalin-fixed paraffin-embedded sections. *J Histochem*

Cytochem. **33**: 737-743.

2. Honda A, Miyazaki T, Iwamoto J, Hirayama T, Morishita Y, Monma T, Ueda H, Mizuno S, Sugiyama F, Takahashi S, and Ikegami T. 2020. Regulation of bile acid metabolism in mouse models with hydrophobic bile acid composition. *J Lipid Res*. **61**: 54-69.
3. Heuman DM. 1989. Quantitative estimation of the hydrophilic-hydrophobic balance of mixed bile salt solutions. *J Lipid Res*. **30**: 719-730.
4. Gao F, Yokoyama S, Fujimoto M, Tsuneyama K, Saiki I, Shimada Y, and Hayakawa Y. 2015. Effect of keishibukuryogan on genetic and dietary obesity models. *Evid Based Complement Alternat Med*. **2015**: 801291.

Supplementary Figure legends

Figure S1 Liver-specific genome-editing analyses using mutant GFP-mice. (A) Analysis of genome-editing efficiency using mutant GFP-mice. AAVs expressing SaCas9 under liver-specific promoter and target gRNA against mutant GFP under human-U6 promoter were injected into 2- to 9-week-old mice. After 2–8-weeks of the injection, the organs were excised and recovery of GFP activity was confirmed by fluorescence microscopy. (B) Schematic diagram of the liver-specific SaCas9-expressing AAV vectors. (C) Comparison of genome-editing efficiency of liver-specific SaCas9-expressing AAV vectors. Mutant GFP-mice (9-week-old) were infected with AAV and livers were analyzed with a fluorescence microscope after 4 weeks of infection. (D and E) Comparison of genome-editing efficiency in the liver and other tissues. Liver-specific genome-editing in juvenile (2-weeks-old) mice was induced by the injection of AAV. After 8 weeks (D) and 2 weeks (E), recovered GFP activities in the liver and other tissues were analyzed. White line, 100 μm.

Figure S2 Ratio of bile acid composition in the liver derived from Abcb4-deficient mice. Ratio of various bile acids in the liver derived from WT and CYPDKO mice with Abcb4 deficiency ($n = 7$ for CYPDKO/NTC mouse livers, $n = 7$ for CYPDKO/Abcb4-KO mouse livers, $n = 10$ for WT/NTC mouse livers, and $n = 11$ for WT/Abcb4-KO mouse livers). Results are represented as mean ± SD (one-way ANOVA, $*P < 0.05$, $**P < 0.01$.).

Table S1 CRISPR/Cas9 target sequences

gRNA for the target gene	Target sequence (without PAM)
EGFP-gRNA	AAGTTCATCTGCACCACCGGC
Mutant GFP-gRNA	AGGGCGAGGAGCTGTTCACCG
Abcb4-gRNA1	TCCGGAACGCAGATGTCATCG
Abcb4-gRNA2	GAAAATATCCGCTATGGCCGT
Abcb4-gRNA3	CTGAAGCACTGGCACGTTGGC

Table S2 PCR primers for detection of mouse gene expression

Mouse genes	Forward primer (5'→3')	Reverse primer (5'→3')	Probe number
Tbp	ggcggtttggctaggttt	gggttatcttcacacaccatga	107
Tnfα	tcttctcattcctgcttgtgg	ggtctgggccatagaactga	49
IL1β	agttgacggaccccaaaag	agctggatgctctcatcagg	38
Tgfβ1	tggagcaacatgtggaactc	gtcagcagccggttacca	72
Col1a1	acctaagggtaccgctgga	tccagcttctccatctttgc	19
Timp1	gcaaagagctttctcaaagacc	agggatagataaacagggaaacact	76
αSma	ctctcttccagccatctttcat	tataggtggtttcgtggatgc	58
Abcb4	gaggtgaagaagggccagac	ctggaccactgtgctcttcc	74
Abcb11	gccacagcaatttgacaccc	ctaccctttgcttctgccca	63
Ntcp	aaggccacactatgtaccctacgtc	gatgctgttgcccacattga	Syber
Mrp2	actggacaagccacaattcc	ctgcaggagtgctcgtatca	Syber
Mdr1a	tagccaacatagcgcgttcc	gttaatgtgtgcgtgtgtgtgc	Syber
Abca1	gagttctttggacttgcgtt	catcatcactttggtccttg	Syber
Abcg5	ggcatgctcaatgctgtgaa	atgatacaggccatcctgactctc	Syber
Abcg8	ggctcaggatcggctttcac	ccttgacacaggcatgaagca	Syber
Fgf15	gctctgaagacgattgccatc	gtagcctaaacagtccatttcctc	TAMRA-FAM
Nr1h4	caaaatgactcaggaggagtacg	tccttgatgtattgtctgtctgg	100
Asbt	tatgggttgctgccctgga	gtgtggagcaagtggtcatgcta	Syber
Cyp7a1	tcaagcaaacaccattcctg	ggctgctttcattgcttca	50
Cyp7b1	aattggacagcttggtctgc	ttctcggatgatgctggagt	99
Cyp8b1	caggaagttccgtcgatttg	ggccccagtagggagtagac	60
Cyp27a1	gcctcacctatgggatcttca	tcaaagcctgacgcagatg	Syber
Hmgcr	caccatgccatcgatagaga	gctccttgaacacctagcatct	77
Srebp1c	ggttttgaacgacatcgaaga	cgggaagtcactgtcttggt	78
ChREBP	ggcctggctggaacagta	cgaagggaattcaggacagt	108

Table S3 liver bile acid composition

**Mouse**		free CA	free ωMCA	free αMCA	free βMCA	free HCA	free CDCA	free DCA	free LCA	free UDCA	free MDCA	free HDCA	free 7oxo-LCA	free 12oxo-LCA	free 12oxo-CDCA
		nmol/whole liver	nmol/whole liver	nmol/whole liver	nmol/whole liver	nmol/whole liver	nmol/whole liver	nmol/whole liver	nmol/whole liver	nmol/whole liver	nmol/whole liver	nmol/whole liver	nmol/whole liver	nmol/whole liver	nmol/whole liver
CYPDKO/NTC	Average	2.737232034	0	0.003992569	0.087103298	0.044347017	2.805552826	1.959712842	1.400852897	4.781867639	0.660364282	0.174675142	0.278489218	0.09655282	0.190541941
CYPDKO/Abcb4-KO	Average	7.774857207	0	0.006478488	0.287326335	0.890901616	8.443415521	1.269187715	1.912057513	10.16039117	0.738520079	0.732941799	1.177297323	0.062484322	0.886799382
WT/NTC	Average	5.187610512	5.721135167	0.749614114	17.27820256	0.025041418	0.20014148	0.252531872	0.106115162	0.478900249	0.529662217	0.206758556	0.028407831	0.049048075	0.397495444
WT/Abcb4-KO	Average	19.30612507	18.99799141	1.583536387	73.23100318	0.111442775	0.863237687	0.340413118	0.144822057	0.483570831	0.404871587	0.24530964	0.060645295	0.065763162	2.02607275
CYPDKO/NTC	STDEV	1.925997064	0	0.007788345	0.117753155	0.074244307	1.04284408	0.634273657	0.523006361	3.460329119	0.279895501	0.069792037	0.341422244	0.053023953	0.289756181
CYPDKO/Abcb4-KO	STDEV	7.931902264	0	0.013839122	0.383546275	1.523716638	7.477931386	0.828270994	1.232283186	9.618963611	0.408530822	0.718952568	1.370070709	0.035342438	1.439226054
WT/NTC	STDEV	3.027533964	2.950504863	0.356162743	11.14767716	0.013968935	0.05003505	0.097091343	0.044996024	0.277499573	0.266369171	0.127709651	0.010789663	0.01347208	0.191190307
WT/Abcb4-KO	STDEV	24.84950294	15.6627661	2.026120203	70.08036216	0.162531677	0.502296098	0.198845979	0.067045801	0.543799062	0.41214842	0.208996055	0.055290254	0.051572734	2.30860191

**Mouse**		free 7oxo-DCA	free 3dehydro-LC	free 3dehydro-D	free 3dehydro-C	free 3dehydro-U	free 3dehydro-C	free 3epi-CA	free 7epi-CA	free 12epi-CA	free 12epi-DCA	free 3epi-CDCA+	free 3epi-UDCA	free 3epi-LCA	total free BA
		nmol/whole liver	nmol/whole liver	nmol/whole liver	nmol/whole liver	nmol/whole liver	nmol/whole liver	nmol/whole liver	nmol/whole liver	nmol/whole liver	nmol/whole liver	nmol/whole liver	nmol/whole liver	nmol/whole liver	nmol/whole liver
CYPDKO/NTC	Average	2.308442168	0.373534296	0.309401149	0.094775472	0.137049829	0.027706529	0.118335965	0.328042917	0.005204275	0.016618721	0.171247471	0.113008489	0.478825712	19.70347752
CYPDKO/Abcb4-KO	Average	9.1143182	0.426582825	0.819431801	0.270161535	0.305466679	0.074728243	0.294246164	0.335520906	0.025717397	0.012858065	0.28948283	0.524793937	0.595460725	47.43142777
WT/NTC	Average	2.619815402	0.006479723	0.06922205	0.076440745	0.059522932	0.077952171	0.018529039	0.259318701	0.00727742	0.014327809	0.018630231	0.014309551	0.008004276	34.46049471
WT/Abcb4-KO	Average	16.58377489	0.00649428	0.053622291	0.028616743	0.068699746	0.154392256	0.090491627	0.598169796	0.023217386	0.026943857	0.016348322	0.021567182	0.007001402	135.5441447
CYPDKO/NTC	STDEV	3.83397122	0.193175851	0.180012556	0.033550397	0.0614698	0.024586536	0.233769787	0.399930986	0.005270006	0.009065316	0.08979912	0.047468646	0.215622543	12.28998647
CYPDKO/Abcb4-KO	STDEV	12.95086366	0.14260393	1.158478847	0.157752493	0.153145971	0.060024787	0.552381082	0.342337782	0.02834555	0.007489481	0.203156177	0.379882153	0.324456783	47.35696145
WT/NTC	STDEV	1.463138031	0.006662429	0.039954747	0.054782436	0.024980466	0.043447385	0.041522737	0.219241722	0.010327994	0.012163279	0.009032169	0.009943266	0.002677624	16.25874671
WT/Abcb4-KO	STDEV	23.95871107	0.003347197	0.044721215	0.016455537	0.026579038	0.120638757	0.158873567	1.173681563	0.033070108	0.031200387	0.010298389	0.019772248	0.003608271	136.2868453

**Mouse**		GCA	GCDCA	GDCA	GLCA	GUDCA	total GBA
		nmol/whole liver	nmol/whole liver	nmol/whole liver	nmol/whole liver	nmol/whole liver	nmol/whole liver
CYPDKO/NTC	Average	0.073820048	0.062359225	0.282754063	0.107608204	0.13204414	0.65858568
CYPDKO/Abcb4-KO	Average	0.045026716	0.143678602	0.064198376	0.07223598	0.114875016	0.440014691
WT/NTC	Average	0.200750527	0.006904281	0.037499386	0.000941802	0.030179315	0.276275311
WT/Abcb4-KO	Average	0.649581057	0.00336661	0.004072693	0.00058969	0.01456849	0.67217854
CYPDKO/NTC	STDEV	0.078142404	0.069100473	0.290794703	0.066086914	0.084991627	0.540415259
CYPDKO/Abcb4-KO	STDEV	0.050198836	0.060168906	0.092713812	0.049143019	0.095820284	0.26287186
WT/NTC	STDEV	0.149049774	0.018871498	0.076627486	0.0027911	0.030216644	0.198284631
WT/Abcb4-KO	STDEV	0.419606596	0.005062194	0.007750387	0.001856567	0.008325497	0.428306997

**Mouse**		TCA	TωMCA	TαMCA	TβMCA	TCDCA	TDCA	TLCA	TUDCA	THDCA	total TBA	all BA
		nmol/whole liver	nmol/whole liver	nmol/whole liver	nmol/whole liver	nmol/whole liver	nmol/whole liver	nmol/whole liver	nmol/whole liver	nmol/whole liver	nmol/whole liver	nmol/whole liver
CYPDKO/NTC	Average	21.02924683	0.216292768	0.097549161	1.315730977	86.64641053	129.065071	29.90233916	44.65564695	1.27769835	314.2059857	334.5680489
CYPDKO/Abcb4-KO	Average	47.20478957	0.700617961	0.144455383	10.7688032	371.7716296	92.21493254	65.58952492	105.3001509	7.997403492	701.6923075	749.56375
WT/NTC	Average	46.06093712	102.7099607	8.638460615	90.18189409	3.570212247	12.47516239	0.107955521	4.735956497	2.235106884	270.7156461	305.4524161
WT/Abcb4-KO	Average	155.5930301	280.1957897	14.22144099	314.1929238	10.28622962	11.74330663	0.05105208	6.617731281	1.84223897	794.7437432	930.9600664
CYPDKO/NTC	STDEV	9.343145916	0.161000799	0.174372472	0.806045193	22.76971317	45.80750982	9.438269613	23.57172492	0.36117308	87.28013623	88.40631303
CYPDKO/Abcb4-KO	STDEV	19.57108415	0.901885079	0.325713325	8.917216058	141.4718057	56.07276884	12.31391809	46.63534494	4.427018895	187.2454572	220.4470763
WT/NTC	STDEV	16.70421175	49.70572707	4.851376579	37.68511846	1.604116737	6.180184653	0.106491053	2.373252266	2.184101127	83.52616013	93.51503613
WT/Abcb4-KO	STDEV	33.84591439	144.8801574	6.538803171	155.3868931	8.351696077	7.363992643	0.08762752	4.229236458	1.547862931	314.5515555	375.0404456

CYPDKO/NTC, n=7; CYPDKO/Abcb4-KO, n=7; WT/NTC, n=10; WT/Abcb4-KO, n=11

Sup Figure 1-1

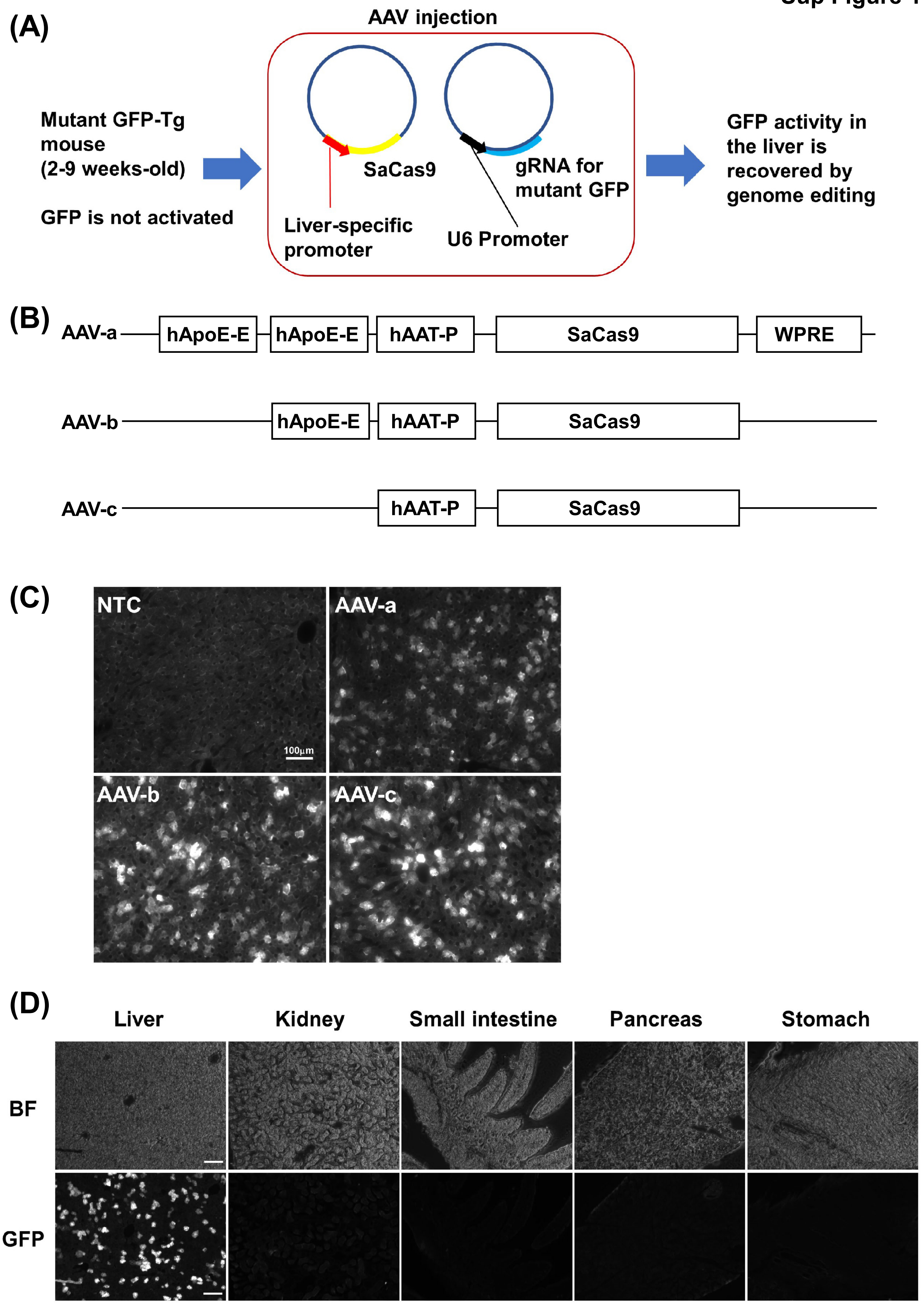

(E)

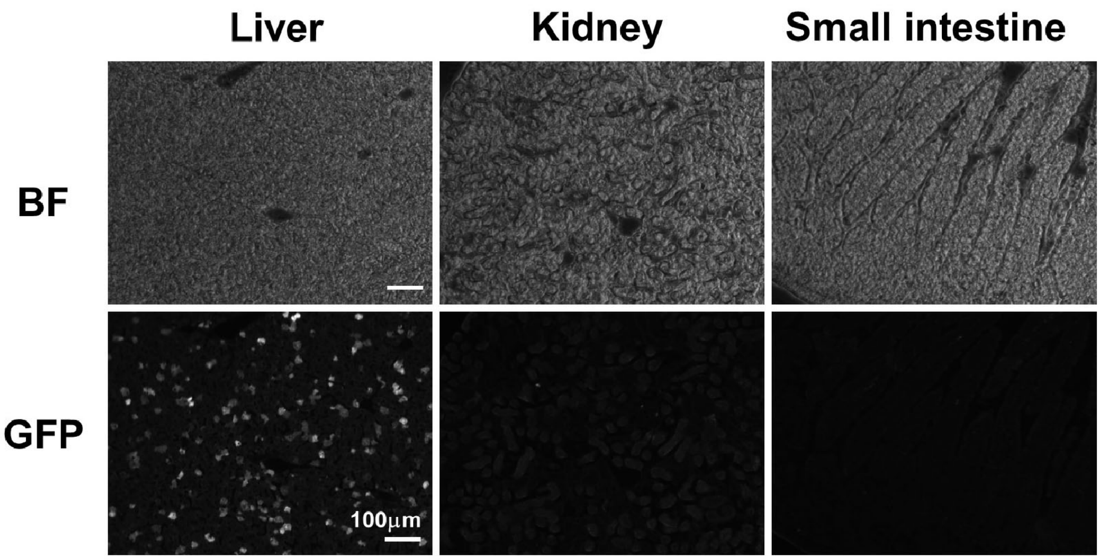

Figure S1 Liver-specific genome-editing analyses using mutant GFP-mice. (A) Analysis of genome-editing efficiency using mutant GFP-mice. AAVs expressing SaCas9 under liver-specific promoter and target gRNA against mutant GFP under human-U6 promoter were injected into 2- to 9-week-old mice. After 2-8-weeks of the injection, the organs were excised and recovery of GFP activity was confirmed by fluorescence microscopy. (B) Schematic diagram of the liver-specific SaCas9-expressing AAV vectors. (C) Comparison of genome-editing efficiency of liver-specific SaCas9-expressing AAV vectors. Mutant GFP-mice (9-week-old) were infected with AAV and livers were analyzed with a fluorescence microscope after 4 weeks of infection. (D and E) Comparison of genome-editing efficiency in the liver and other tissues. Liver-specific genome-editing in juvenile (2-weeks-old) mice was induced by the injection of AAV. After 8 weeks (D) and 2 weeks (E), recovered GFP activities in the liver and other tissues were analyzed. White line, 100 µm.

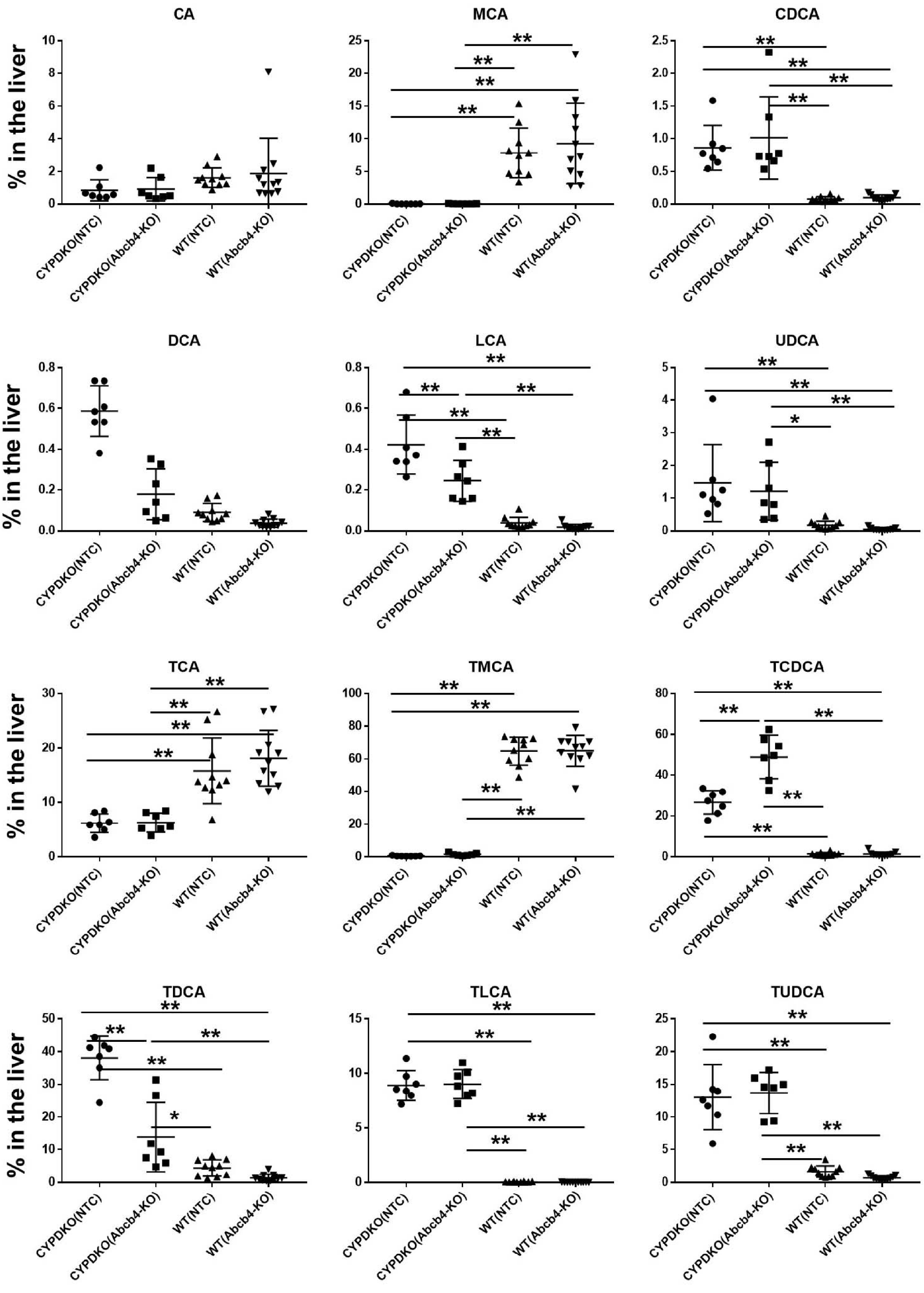

Figure S2 Ratio of bile acid composition in the liver derived from Abcb4-deficient mice. Ratio of various bile acids in the liver derived from WT and CYPDKO mice with Abcb4 deficiency (n = 7 for CYPDKO/NTC mouse livers, n = 7 for CYPDKO/Abcb4-KO mouse livers, n = 10 for WT/NTC mouse livers, and n = 11 for WT/Abcb4-KO mouse livers). Results are represented as mean ± SD (one-way ANOVA, *P < 0.05, **P < 0.01.).
